# Editorial: New insights into the influences of soil nutrients on plant-fungal symbiosis in agro- and forest ecosystems

**DOI:** 10.3389/fmicb.2023.1237534

**Published:** 2023-07-18

**Authors:** Kai Sun, Fang-Dong Zhan, Yu Shi, Jiayu Zhou, Jun Zhou, Long Peng

**Affiliations:** ^1^Jiangsu Key Laboratory for Microbes and Functional Genomics, Jiangsu Engineering and Technology Research Center for Industrialization of Microbial Resources, College of Life Sciences, Nanjing Normal University, Nanjing, Jiangsu, China; ^2^College of Resources and Environment, Yunnan Agricultural University, Kunming, China; ^3^State Key Laboratory of Crop Stress Adaptation and Improvement, School of Life Sciences, Henan University, Kaifeng, China; ^4^Henan Dabieshan National Field Observation and Research Station of Forest Ecosystem, Zhengzhou, China; ^5^Xinyang Academy of Ecological Research, Xinyang, China; ^6^Institute of Botany, Jiangsu Province and Chinese Academy of Sciences, Nanjing, China; ^7^Chrono-Environnement UMR 6249, CNRS, Université Bourgogne Franche-Comté, Besançon, France; ^8^Research Institute of Subtropical Forestry, Chinese Academy of Forestry, Hangzhou, China

**Keywords:** soil nutrient availability, nutrient forms, symbiotic fungi, arbuscular mycorrhizal fungi, fungal endophyte, fungal community, plant-fungal symbiosis, agroecosystem and forest ecosystem

Symbiotic fungi are widely distributed and form associations with over 90% of terrestrial plant species, playing an essential role in global agro- and forest ecosystems (Behie and Bidochka, 2014). These symbionts provide various benefits to host plants, including increased biomass accumulation (Hiruma et al., 2016), enhanced nutrient uptake (Guether et al., 2009; Fochi et al., 2017), and improved environmental adaptation (Sui et al., 2019). In return, host plants offer suitable habitats and accessible photosynthate for fungal survival and reproduction (Siegel et al., 1987; Jiang et al., 2017). However, in natural ecosystems, plant-fungal symbioses often face changing soil conditions, particularly variations in nutrient status (Sun et al., 2020, 2022). Different nutrient availabilities, forms, and compositions can significantly affect plant metabolism, growth, and immunity, which are crucial for maintaining the interplay between plants and fungi (Saikkonen et al., 2004; Sánchez-Bel et al., 2018). Conversely, soil nutrient status can influence the community composition of soil fungi, thereby impacting the establishment of symbionts.

The goal of this Research Topic is to showcase the latest studies focusing on the global effects of soil nutrients on plant-fungi interactions, enhancing our understanding of the mechanisms underlying the colonization and community dynamics of symbiotic fungi during their interaction with host plants across various species/genotypes, soil types, and nutrient levels and forms. The Research Topic covers studies on arbuscular mycorrhizal fungi (AMF), fungal endophytes and the plant-associated fungal community.

## Insights into the impact of soil nutrients on AMF symbiosis

Ancient AMF symbioses can be seen as the bridge between plants and soils (Saia and Jansa, 2022; Kuyper and Jansa, 2023). The AMF symbionts are influenced by the forms and levels of nutrients in the soil, including nitrogen (N), phosphorus (P), and potassium (K). Yu et al. pointed out that AMF are sensitive to N addition over short timescales (1 year). Beneficial AMF associations are promoted under N-deficient conditions (Bonfante and Genre, 2010; Sanchez-Bel et al., 2016; Sánchez-Bel et al., 2018). Additionally, ammonium reduced AMF colonization levels in numerous plant species compared to nitrate (Pattinson et al., 2000).

Two noteworthy perspectives highlight the important roles of AMF symbiosis in plant invasion in agro-ecosystems based on greenhouse experiments. Du et al. revealed that the AMF *Septoglomus constrictum* provides host plants with higher N and P accumulation, conferring invasive plants with greater advantages over native congeners. Chen et al., through the analysis of AMF community associated with the invasive species *Solidago canadensis* and its native congener *S. decurrens*, clarified that AMF could confer invasive plants with greater advantages over native congeners, dependent on the forms of P in the soil.

Inorganic orthophosphate (Pi) is the available form of P that plants can acquire and utilize. However, Pi is often insufficient in the field due to its low solubility and relative immobilization (Nagy et al., 2009). Under P starvation, AMF can efficiently promote Pi uptake and homeostasis in host plants (Dierks et al., 2021). In this Research Topic, Zhang et al. identify a HLH domain containing transcription factor, *RiPho4*, from *Rhizophagus irregularis*. Through subcellular localization, yeast one-hybrid experiments, and using virus-induced gene silencing approaches, the authors demonstrated that *RiPho4* acts as a transcriptional activator in AMF to maintain arbuscule development and regulate Pi uptake in host plants during Pi starvation. This study provides new insights into the mechanisms underlying how AMF regulates Pi uptake in host plants under Pi deficiency.

Apart from N and Pi, the AMF-plant interaction is also sensitive to changes in soil K nutrient (Han et al., 2023). In a greenhouse experiment, Yuan et al. investigated sweet potato (*Ipomoea batatas* (L.) Lam.]), a versatile crop with high K requirements for enhanced yield. Their study showed that K application and the presence of AMF *Claroideoglomus etunicatum* exhibited a synergistic effect on the root development and K acquisition of the “Xu28” variety of sweet potato, which has high K use efficiency, resulting in significant yield promotion. These results, combined with previous studies, expand our knowledge of the influences of soil nutrients on plant-AMF symbiosis.

## Impact of heavy metal contamination on plant-fungal interactions

Heavy metal contamination in soil is a pressing global issue (Marrugo-Negrete et al., 2017) that affects plant-fungal interactions (Motaharpoor et al., 2019). Dark septate endophytes (DSEs) are ubiquitous colonizers of plant roots in various terrestrial ecosystems, often found in stressful environments, especially heavy metal-polluted soils (Su et al., 2021). Wang et al. highlighted the ability of *Exophiala pisciphila* H93, a beneficial dark septate endophyte which colonizes maize roots, to withstand cadmium exposure without compromising its growth-promotion effect on maize. They found that H93 colonization enhances plant resistance to heavy metal by influencing the expression of genes involved in signal transduction, hormonal pathway, and glutathione metabolism.

## Influence of soil nutrients on plant-associated fungal communities

The response of the plant-associated fungal community to global changes plays a crucial role in understanding carbon and N cycling processes in agro- and forest ecosystems (Falkowski et al., 2008). In this Research Topic, Yu et al. demonstrated that the interaction between N addition and rainfall patterns has significant effects on soil fungal diversity in a grassland ecosystem. Similarly, Zhao et al. showed in a forest ecosystem that climate factors, such as temperature and precipitation, significantly influence dominant fungal genera and functional guilds in soil, particularly ectomycorrhizal fungi. Furthermore, fungal diversity and composition are strongly influenced by seasonal variation in soil nutrients, including total N and available P. The effect of vegetation type on rhizosphere microbes is also of great interest across different ecosystems. Liu et al. compared fungal communities in the rhizosphere of three typical vegetation types (herb, shrubs, and arbors). They discovered that the fungal community structure in the rhizosphere can vary across vegetation types and is primarily governed by deterministic processes. Additionally, plant metabolism has a significant impact on the plant-associated microbiome (Trivedi et al., 2020). Zhu et al. demonstrated that by applying exogenous salicylic acid, beneficial rhizosphere microorganisms were selectively enriched to enhance watermelon resistance to *Fusarium* wilt. These community analyses provide insights into the potential of plant-associated fungi in sustainable agriculture.

In this Research Topic, the majority of the studies aimed to elucidate the influences of changing environmental conditions, particularly soil nutrients, on plant-fungal symbiosis. However, more efforts are needed to clarify the molecular mechanisms underlying how nutrient factors drive the maintenance and breakdown of plant-fungi symbionts. Additionally, limited studies have been conducted under field conditions, which could provide better guidance for agricultural production. We anticipate further novel discoveries in the future.

## Author contributions

KS wrote the manuscript. All authors reviewed and revised the manuscript.
